# Experimental Study on the Upstream Migration Behavior of Adult *Leptobotia elongata* Under Flow Heterogeneity and Schooling in a Controlled Flume System

**DOI:** 10.3390/ani16081266

**Published:** 2026-04-20

**Authors:** Lixiong Yu, Jiaxin Li, Fengyue Zhu, Min Wang, Yuliang Yuan, Huiwu Tian, Mingdian Liu, Weiwei Dong, Majid Rasta, Chunpeng Bao, Shenwei Zhang, Xinbin Duan

**Affiliations:** 1 National Agricultural Science Observing and Experimental Station of Chongqing, Yangtze River Fisheries Research Institute, Chinese Academy of Fishery Science, Wuhan 430233, China; yulixiong@yfi.ac.cn (L.Y.);; 2 State Key Laboratory of Hydraulics and Mountain River Engineering, Sichuan University, Chengdu 610065, China; 2020223060060@stu.scu.edu.cn; 3 Institute of Hydroecology, Ministry of Water Resources and Chinese Academy of Sciences, Wuhan 430079, China; dongww@mail.ihe.ac.cn; 4 College of Hydraulic and Environment Engineering, China Three Gorges University, Yichang 443002, Chinachunpengbao@163.com (C.B.);

**Keywords:** *Leptobotia elongata*, potamodromous fish, swimming behavior, probability density distribution, fishway

## Abstract

Fishways play a critical role in restoring river connectivity and conserving fishery resources, yet their efficiency is often limited by mismatches between hydraulic conditions and species-specific behavioral traits. To quantify the upstream migration behavior of fish under the combined influence of flow heterogeneity and schooling effects, this study examined the endangered species *L. elongata* in the Yangtze River Basin. Volitional swimming behavior was tested in an open-channel flume under three spatially heterogeneous flow regimes (I: Low–Moderate–High; II: High–Moderate–Low; III: Moderate–High–Low). A video monitoring system recorded the upstream movement of solitary fish and three-individual schools. Swimming trajectories, upstream migration time, preferred flow velocities, and schooling metrics—including nearest neighbor distance (NND) and mean pairwise distance (MPD)—were analyzed. Linear mixed-effects models were employed to account for repeated measures and individual variability. Results showed that schooling behavior significantly enhanced upstream migration efficiency: schooling fish arrived at the target area on average 8.93 s earlier than solitary individuals (*p* < 0.01), while flow condition alone had no detectable effect on arrival time. *L. elongata* consistently preferred low-velocity zones (0.20–0.50 m/s) and avoided high-velocity regions (0.75–1.25 m/s), with meandering upstream trajectories predominating. NND showed no significant differences across flow conditions (*p* > 0.05), indicating stable schooling cohesion. However, MPD increased significantly under Flow III compared to Flows I and II (*p* < 0.01), suggesting that higher flow heterogeneity leads to more dispersed group spacing while overall cohesion is maintained. Distinct movement strategies were observed: solitary fish predominantly utilized boundary regions as hydraulic refuges (wall-following: 63.8–80.5%), whereas schools exhibited greater spatial exploration and reduced wall-following. These findings demonstrate that schooling enhances migration efficiency while preserving a cohesive group structure and that flow heterogeneity influences within-group spatial organization. To optimize fishway performance for *L. elongata*, we recommend maintaining flow velocities within 0.20–0.50 m/s. This study provides scientific guidance for hydraulic regulation in fishway design and habitat restoration, emphasizing the combined effects of flow heterogeneity and schooling behavior on migration performance.

## Simple Summary

The absence of information on species-specific preferred velocities and upstream schooling behavior can affect fishway efficiency. In this study, the upstream swimming behavior of an endangered fish (*Leptobotia elongata*) was studied in a laboratory flume under different flow velocity conditions, comparing solitary individuals with small schools. Schooling fish reached the upstream area significantly faster (by 8.93 s on average) than solitary fish. Both solitary and schooling fish preferred low-velocity zones (0.20–0.50 m/s) and avoided high-velocity areas. Schools maintained a cohesive group structure across all flow conditions, although group spacing increased under higher flow heterogeneity. Solitary fish mainly swam near walls, while schools explored a wider range of the channel. These findings provide practical guidance for designing fishways that better accommodate this species’ behavior and swimming preferences.

## 1. Introduction

Hydropower development brings substantial socio-economic benefits but also causes significant adverse impacts on aquatic ecosystems and organisms within river basins [1,2,3]. In particular, dam construction disrupts the longitudinal connectivity of rivers, obstructing fish migration routes that are essential for completing life cycles. To mitigate these impacts, fishways are widely constructed to facilitate upstream and downstream fish migration [4,5]. However, numerous studies have shown that the passage efficiency of many operational fishways remains suboptimal, primarily due to mismatches between the hydraulic environment within fishways and the ecological requirements of target fish species, including difficulties in rapidly locating fishway entrances [6,7]. Preferred flow velocity refers to the range of flow velocities that fish actively select during swimming, which can minimize energy expenditure and enhance migration efficiency [8,9,10]. In natural riverine environments, flow conditions are inherently spatially heterogeneous, and fish must continuously adjust their swimming behavior to cope with coexisting velocity gradients. Moreover, fish rarely migrate as isolated individuals; instead, they often exhibit varying degrees of schooling behavior. Therefore, understanding upstream migration requires consideration of the combined effects of flow velocity and schooling behavior to more accurately reflect fish movement characteristics under spatially heterogeneous hydraulic conditions [11,12].

A substantial body of research has investigated fish behavioral responses to flow stimuli. Ouyang et al. (2025) [13] examined the hydrodynamic conditions and swimming behaviors of *Schizothorax prenanti* in fishways with three vertical-slot configurations using laboratory experiments and numerical simulations, demonstrating that slot geometry strongly influences flow structure and passage efficiency. At smaller hydrodynamic scales, Wang et al. (2025) [14] quantified the effects of wake vortices generated by obstacles of different sizes on fish propulsion and swimming stability, while Tang et al. (2024) [15] further examined fish entrainment within vortices formed in the wake of semi-cylindrical structures. Recent studies have also highlighted the importance of schooling behavior under high-velocity and turbulent conditions. Zhang et al. (2024) [16] showed that giant danio (*Devario aequipinnatus*) swimming in schools under elevated flow velocities and turbulence levels reduced total energy expenditure by 63–79% compared with solitary individuals. Schooling fish maintain swimming efficiency by reducing group volume without increasing tail-beat effort, indicating that collective behavior can effectively mitigate adverse hydraulic effects.

Schooling, as a fundamental biological trait of fish, plays a critical role in fishway passage efficiency [17,18]. Schooling fish exhibit enhanced navigational capabilities, enabling them to locate migration routes more rapidly, reduce migration time, and increase migration success rates [19,20]. During upstream migration, fish density not only affects the ability of fish to locate fishway entrances but also influences upstream motivation and average transit time [21]. These effects are closely associated with individual behavioral responses to the positions and movements of neighboring conspecifics within the school [22,23]. While energy-saving strategies govern space use in flowing waters regardless of group size, exploration and swimming activity increase with school size, although the magnitude of this effect is modulated by flow velocity [8,24]. As school size increases from two to eight individuals, fish show an increasing tendency to synchronize swimming speed with the group [25]. When a school enters a fishway and encounters spatially varying flow velocities, interactions among individuals gradually begin to govern collective upstream migration behavior [26]. Despite these advances, existing knowledge remains insufficient in several key aspects. Most studies on fish migration have focused on solitary individuals under uniform or quasi-uniform flow conditions, which oversimplify the spatially heterogeneous hydraulic environments characteristic of natural rivers and engineered fishways. Consequently, these studies may not adequately capture how fish perceive and respond to coexisting velocity gradients during upstream movement. In particular, it remains unclear how migratory fish, especially endangered species, successfully negotiate upstream migration through spatially heterogeneous flow fields while exploiting the benefits of schooling behavior.

**Figure 1:**
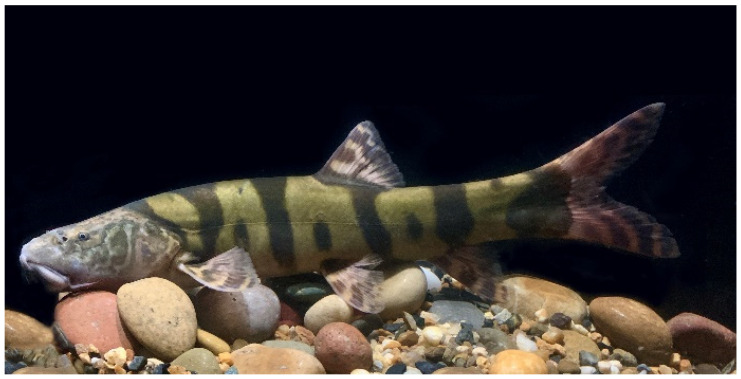
*Leptobotia elongata*.

*L. elongata* is a migratory fish endemic to the upper Yangtze River (Figure 1), belonging to the typical riverine drifting-egg spawners whose natural reproduction is highly dependent on suitable hydrological conditions, particularly flow velocity stimulation. As a migratory river fish, *L. elongata* typically undertakes short-distance upstream migrations along river channels during the reproductive season to access spawning and feeding grounds, thereby completing its life cycle. Physiologically, this species exhibits morphological and functional adaptations to flowing-water environments, including an elongated body shape and strong station-holding and sustained swimming abilities. Behaviorally, *L. elongata* shows pronounced rheotactic responses and frequently forms small groups during upstream movement, which may enhance navigational efficiency under spatially heterogeneous flow velocities. Its upstream migratory activity is seasonally constrained, occurring primarily during periods of elevated discharge and suitable water temperatures during the reproductive season [27]. However, the cascade hydropower development in the upper Yangtze River has severely degraded the riverine habitats upon which this species depends. Dam construction has disrupted river longitudinal connectivity, blocking access to historical spawning grounds and inhibiting the natural reproductive activities of *L. elongata*. As a consequence of hydropower development in the upper Yangtze River, this species has experienced a sharp population decline and is therefore listed as Vulnerable (VU) in the *Red Data Book of Endangered Animals in China*, presenting substantial challenges for species conservation and resource restoration [28]. To mitigate the impacts of hydropower development on fish resources in the Yangtze River, fish passage facilities have become essential ecological compensation measures. However, existing fishway designs often lack an in-depth understanding of the ecological requirements of target species, particularly the upstream migration patterns of endemic species like *L. elongata*, resulting in a gap between expected and actual fish passage performance. Therefore, investigating the upstream migration behavior and hydraulic response mechanisms of *L. elongata* holds important scientific value and practical significance for optimizing fishway hydraulic design, improving fish passage efficiency, and promoting species conservation.

This study focuses on the volitional swimming behavior of *L. elongata* in an open-channel flume under spatially heterogeneous flow velocities. The primary objectives of this study are to (i) quantify the preferred flow velocity of *L. elongata*, (ii) elucidate upstream swimming behavior under different spatially heterogeneous flow conditions, and (iii) evaluate the influence of schooling condition on autonomous swimming behavior in an open channel flume. The findings of this study have broad implications not only for the design and optimization of fishways for target species but also for providing a novel experimental framework applicable to other potamodromous migratory fish.

## 2. Materials and Methods

### 2.1. Experimental Fish

Adult *L. elongata* (body length: mean ± SD = 20.67 ± 1.78 cm; body mass: mean ± SD = 209.55 ± 24.08 g; *n* = 30) were obtained from the Jingzhou Breeding Base of the Yangtze River Fisheries Research Institute, Chinese Academy of Fishery Science (Animal Welfare and Ethics Approval Number: YFI2026005), on 25 June 2025. Fish were reared in a recirculating holding tank (3000 L), sterilized with potassium permanganate to minimize the risk of disease. Water temperature was maintained at 23.0 ± 1.0 °C using a thermostatic control system, dissolved oxygen was kept above 7.0 mg/L, and approximately 20% of the total water volume was renewed daily. To minimize the effects of light on *L. elongata*, shading cloth was used to reduce light exposure. Experimental fish were fed to apparent satiation once daily with the same commercial diet (submerged feed; protein content 35%; Tongwei Group, China). Uneaten feed and feces were removed by siphoning 30 min after feeding.

### 2.2. Experimental Apparatus

**Figure 2:**
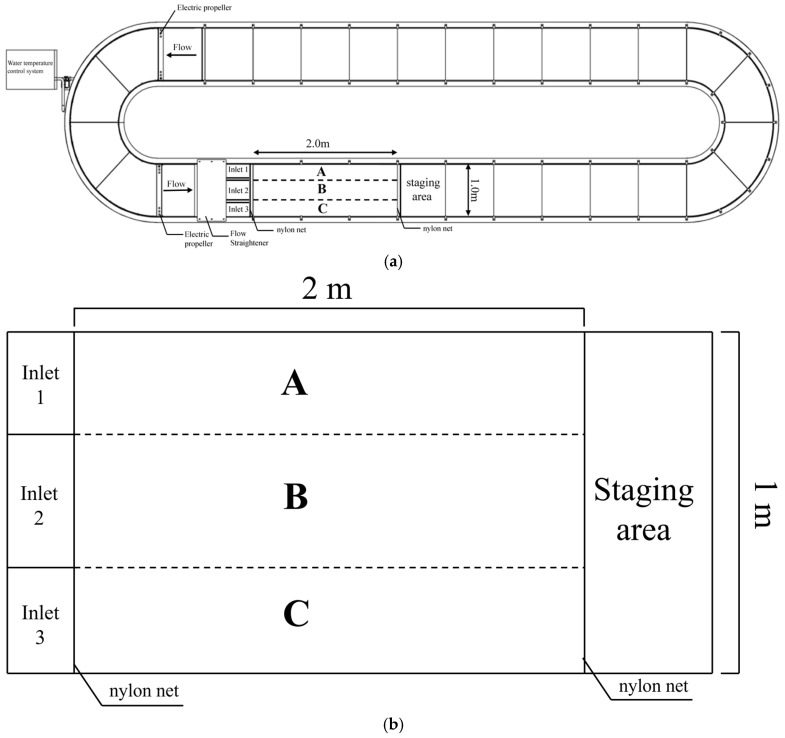
(**a**) Self-designed open recirculating variable-frequency flume: electric propeller, flow straightener, retention screen, swim test area, staging area, and water temperature control system; (**b**) experimental working sector.

The experiments were conducted in a self-designed, open, recirculating, variable-frequency flume (Figure 2). The experimental section was 2.0 m long and 1.0 m wide and was designed to ensure uniform hydraulic conditions and consistent behavioral observations across treatments. Water flow was generated using an electric propeller, with flow velocity regulated by a variable-frequency drive controlling the propeller speed (thrust: 180 lbf; power: 2700 W; Ningbo Youdong Electric Motor Co., Ltd., Ningbo, China). Upstream flow-straightening grids with varying densities were installed to generate three distinct flow velocity zones—low, moderate, and high—in the downstream experimental section, labeled A, B, and C, respectively, each with a width of 0.33 m. Two nylon nets with a mesh size of 10 mm were installed at the upstream and downstream ends of the test section to prevent fish escape. The nets did not obstruct flow, and fish were free to turn or hold position upon contact. An infrared network camera (DS-2CD2T87SWDV2-L, Hikvision, China) was mounted 6.0 m above the flume to record the swimming behavior of *L. elongata*.

### 2.3. Experimental Hydraulic Parameters

The experimental velocities were set above the rheotaxis threshold but below the critical swimming speed of *L. elongata* to ensure uninterrupted upstream swimming and allow behavioral responses to different flow velocities to be characterized. The average induction velocity of *L. elongata* was 0.15–0.18 m/s, the average critical swimming speed was 0.78–1.09 m/s, and the average burst swimming speed was 1.10–1.58 m/s (Table 1). Prior to the experiment, a current meter (LS45A; measurable velocity range: 0.015–3.5 m/s; operating water depth: 0.05–3 m; relative mean square deviation ≤ 2%; Chongqing Hydrological Instrument Factory, Chongqing, China) was used to measure the flow velocity at the flume inlet, ensuring that the velocities under the three experimental conditions were approximately 0.50, 0.80, and 1.20 m/s, respectively. During the experiments, the propeller operated at 80.0 ± 3.7 rpm, water depth was maintained at 45.0 ± 0.3 cm, and water temperature was controlled at 24.0 ± 0.5 °C using a temperature regulation system (KH430S YIE/A, Guangdong Tongyi Heat Pump Science and Technology Co., Ltd., Guangzhou, China). All experiments were conducted in the straight section of the indoor open flume, where an unobstructed flow field was generated by adjusting the variable-frequency drive. After the experiment, the experimental arena was subdivided into 0.10 m × 0.10 m grids, resulting in a total of 200 discrete sampling units. Flow velocity within each grid was measured using the same current meter for subsequent analysis.

### 2.4. Experimental Setup

Experiments were conducted in an open-channel flume between 3 and 21 July 2025 during daylight hours (9:00 am to 16:00 pm), with each trial lasting 80 min. For each trial, *L. elongata* individuals were randomly selected from the holding tank and transferred to the staging area, where they acclimated to ambient temperature and flow conditions for 1 h. After acclimation, movable partitions were lifted, allowing fish to swim freely across the experimental area for 20 min (Figure 2). Following each trial, observed fish were transferred to one of three temporary holding tanks (3000 L) labeled according to the experimental condition to prevent repeated measurement under the same condition. Due to the limited number of fish, individuals could be reused across different experimental conditions. School size was set to three fish, sufficient to elicit schooling behavior [29], and school trials followed the same procedures. To reduce potential effects of prior experience and partially maintain the independence of observations, a minimum interval of 1 day was maintained between successive trials [30]. Each experimental condition was replicated five times.

### 2.5. Statistical Analysis

The experimental data were preprocessed using Microsoft Excel 2018 and analyzed statistically with R 4.3.3. Linear mixed-effects models (LMMs) were employed to examine differences in upstream migration efficiency, nearest neighbor distance (NND), and mean pairwise distance (MPD) under different experimental conditions while accounting for repeated measurements and the non-independence among individual fish. For upstream migration efficiency, the first-arrival time was specified as the response variable. Fixed effects included Condition (solitary vs. schooling fish) and Flow (flow conditions I, II, and III), and FishID was included as a random intercept to account for repeated measures within individuals. An initial model including the interaction between Condition and Flow was fitted and compared to a main-effects-only model using likelihood ratio tests based on maximum likelihood (ML) estimation. When the interaction was not significant, the more parsimonious main-effects model was selected. Model parameters were estimated using restricted maximum likelihood (REML). Residual diagnostics, including quantile–quantile plots and residual vs. fitted plots, were performed to assess the assumptions of normality and homoscedasticity. Effect sizes and 95% confidence intervals for fixed effects were calculated and reported. Preferred velocity curves were generated using the *ggplot2* (version 3.5.2) package. For nearest neighbor distance (NND), the response variable was log-transformed to improve normality and reduce skewness (*log*-*Dist* = *log* (NND + 0.001)). A linear mixed-effects model was fitted with Flow as a fixed effect and FishID as a random intercept to account for repeated measurements of individual fish. Model parameters were estimated using REML, and residual diagnostics were conducted to assess normality and homoscedasticity. The significance of fixed effects was tested using Type II *ANOVA* (version 1.1-37) with Satterthwaite’s approximation. Pairwise comparisons among flow levels were performed using Tukey-adjusted estimated marginal means with the *emmeans* (version 2.0.2) package. For mean pairwise distance (MPD), MPD was calculated for each three-fish group at 10 s intervals as the average Euclidean distance between all pairs of individuals within the group. Linear mixed-effects models were fitted with Flow as a fixed effect and GroupID as a random intercept to account for repeated measurements within each group. Time was standardized prior to analysis to improve model convergence. Model parameters were estimated using REML, and residual diagnostics were conducted to assess normality and homoscedasticity. The significance of fixed effects was tested using Type III *ANOVA* (version 1.1-37) with Satterthwaite’s approximation, and pairwise comparisons among flow levels were performed using *Tukey*-adjusted estimated marginal means with the *emmeans* (version 2.0.2) package. Statistical results are reported as *Mean* ± *SD*, with *p* < 0.05 considered significant.

#### 2.5.1. Tracking of Swimming Paths

SwisTrack (version 4.0.0; an open-source video tracking software, Switzerland) was used to determine the two-dimensional locations of *L. elongata* during movement. Consecutive video frames (25 fps) were analyzed, and the trajectories of *L. elongata* were extracted by connecting their positions in chronological order. The frequency of position coordinates within each grid cell and the selection of upstream paths were recorded. Surfer 15 was then used to plot scatter diagrams of fish distribution and upstream path, thereby reflecting the overall movement patterns of the experimental fish within the flume.

#### 2.5.2. Quantification of Upstream Migration Efficiency

Experimental fish were tracked frame by frame using SwisTrack software. The time required for individuals in different experimental groups to reach the inlet for the first time was recorded. For schools, the first-arrival time was defined following Wang et al. (2025) [31], with minor modifications. In this study, the first-arrival time was defined as the time elapsed from the start of the trial until the earliest individual in the three-fish groups reached the inlet. First-arrival times of solitary fish and schools were then compared, and the elapsed time was quantified to characterize upstream migration efficiency, thereby reflecting the short-term migration rate of the experimental fish.

#### 2.5.3. Preferred Velocity Analysis

The coordinates of *L. elongata* from different experimental groups were extracted using Python 3.8.0 (64-bit) with *pandas* (version 1.3.3), *numpy* (version 1.21.6), *matplotlib.pyplot* (version 3.3.1), *scipy.interpolate* (version 1.7.3), *matplotlib.cm* (version 3.3.1), *griddata*, and *interp2d*. The background flow field at the fish-occupied locations was then interpolated using the kriging method. Flow velocity values along the upstream trajectories of the fish were categorized into discrete intervals with a width of 0.33 m. For each interval, the ratio of the area occupied by the fish to the total area of all selected intervals was calculated and compared with the corresponding proportion of the same interval in the background flow field. A positive difference was interpreted as a preference for that velocity, a zero difference as no selection, and a negative difference as avoidance [32].

#### 2.5.4. Nearest Neighbor Distance (NND) Calculation

**Figure 3:**
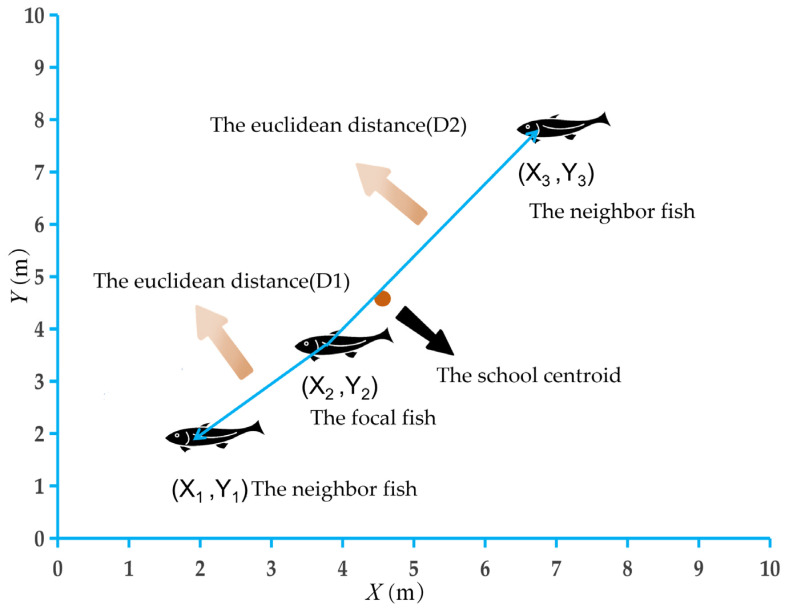
Diagram of the distribution of fish schools.

The nearest neighbor distance (NND) was used to quantify the spatial cohesion of fish groups. For each time frame, the NND was defined as the Euclidean distance between a focal fish and its closest conspecific based on their centroid positions. The distance was calculated for each individual in the group, and the mean NND was obtained by averaging across all individuals and frames within a trial (Figure 3).

(1) $${\mathrm{NND}}_{i}=\underset{j\neq i}{\min}\sqrt{{\left(x_{i}-x_{j}\right)}^{2}+{\left(y_{i}-y_{j}\right)}^{2}}$$

*x_i_* and *y_i_* represent the coordinates of the focal fish *i*, *x_j_* and *y_j_* represent the coordinates of the other fish *j* in the group (*j* ≠ *i*), and the minimum value of the calculated distances represents the nearest neighbor distance.

#### 2.5.5. Mean Pairwise Distance (MPD) Calculation

To quantify group spacing, the mean pairwise distance (MPD) was calculated for each three-fish group at 10 s intervals. MPD represents the average Euclidean distance between all pairs of individuals within a group and reflects the cohesion and spatial structure of the school. For a group of *N* individuals, MPD is defined as

(2) $$\mathrm{MPD}=\frac{2}{N(N-1)}\sum_{i<j}d_{ij}$$

where *d_ij_* is the distance between individuals *i* and *j*. In the present study, each three-fish group had three pairwise distances, so MPD was calculated as the arithmetic mean of these three distances.

## 3. Results

### 3.1. Swimming Path

**Figure 4:**
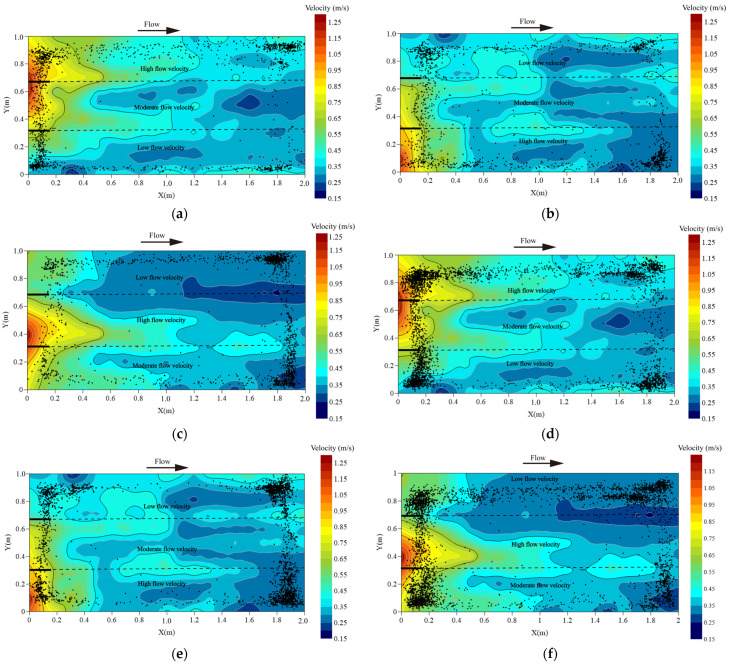
Fish upstream routes (black dots represent the distribution trajectories of the experimental fish): (**a**) solitary fish swimming path in spatially heterogeneous flow velocity I; (**b**) solitary fish swimming path in spatially heterogeneous flow velocity II; (**c**) solitary fish swimming path in spatially heterogeneous flow velocity III; (**d**) schooling group swimming path in spatially heterogeneous flow velocity I; (**e**) schooling group swimming path in spatially heterogeneous flow velocity II; (**f**) schooling group swimming path in spatially heterogeneous flow velocity III.

Significant differences in movement distribution were observed between solitary *L. elongata* and schools under the three spatially heterogeneous flow conditions (Figure 4). Solitary fish predominantly exhibited upstream wall-following behavior. The proportion of wall-following behavior occurring within 0.1 m of the wall was 80.5%, 63.8%, and 66.1% under Conditions I, II, and III, respectively. As shown in Figure 4a–c, individuals were mainly distributed along the flume boundaries and rarely entered the central high-velocity zones. After encountering high-velocity regions, fish typically drifted into adjacent low-velocity areas within 3–7 s, indicating active use of boundary regions as hydraulic refuges. This spatial pattern suggests an energy-conservation strategy, whereby solitary fish reduce locomotor costs while maintaining upstream orientation.

In contrast, schooling fish showed substantially weaker wall-following behavior, with proportions of 23.9%, 20.5%, and 26.7% under Conditions I, II, and III, respectively. Instead, schools displayed more meandering upstream trajectories near the inlet, with tortuosity values of 0.82, 1.83, and 1.60 for the three conditions. The trajectories shown in Figure 4d–f illustrate that groups occupied a wider portion of the channel compared with solitary fish. Under flow conditions I and III, schools overlapped more with the main current and aggregated in higher-velocity regions (Figure 4d,f), suggesting that group movement facilitated exploration and utilization of faster-flow areas. Under Condition II, schools concentrated near the downstream boundary of the low-velocity zone (Figure 4e), indicating that groups maintained cohesion while positioning themselves in hydraulically favorable areas.

Overall, these results indicate distinct upstream movement strategies between solitary and schooling fish in heterogeneous flows: solitary individuals primarily utilize boundary refuges to minimize energy expenditure, whereas schools exhibit greater spatial exploration and can exploit a broader range of hydraulic conditions.

### 3.2. Upstream Migration Efficiency

**Figure 5:**
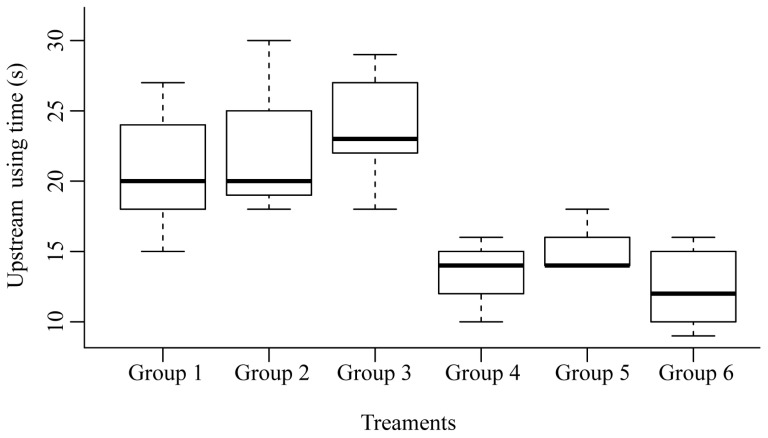
Upstream efficiency comparison. Group 1: spatially heterogeneous flow velocity I for solitary fish; Group 2: spatially heterogeneous flow velocity II for solitary fish; Group 3: spatially heterogeneous flow velocity III for solitary fish; Group 4: spatially heterogeneous flow velocity I for schooling group; Group 5: spatially heterogeneous flow velocity II for schooling group; Group 6: spatially heterogeneous flow velocity III for schooling group.

The linear mixed-effects analysis indicated no significant interaction between social condition (Condition) and flow (Flow) on first-arrival time (*χ*^2^ = 0.76, *df* = 2, *p* = 0.68). Given the lack of interaction, a more parsimonious main-effects model was selected. The main-effects model revealed a significant effect of social condition: schooling fish arrived at the target area significantly earlier than solitary fish (*Estimate* = −8.93, *p* < 0.01), whereas flow velocity had no detectable effect on arrival time (*p* > 0.05) (Figure 5). Substantial variation among groups was observed, as reflected by the GroupID random intercept variance (variance = 9.82). Residual diagnostics confirmed that the model assumptions of normality and homoscedasticity were reasonably satisfied, supporting the validity of the model. Biologically, this suggests that while social context strongly influences the timing of upstream movement—likely through collective facilitation—individual traits or behavioral tendencies also contribute to variability in arrival patterns, independent of flow conditions.

### 3.3. Preferred Velocity

**Figure 6:**
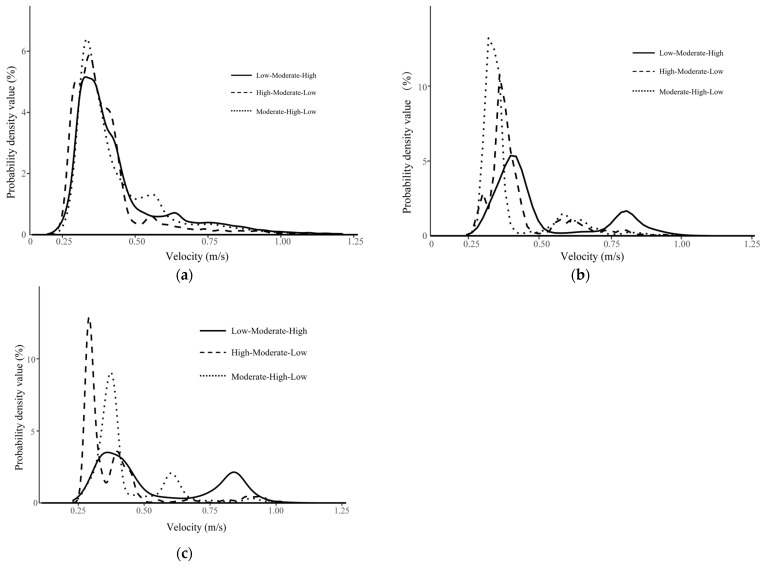
Probability density distribution: (**a**) background flow velocity, (**b**) background flow velocities corresponding to solitary fish trajectory points, and (**c**) background flow velocities corresponding to schooling group trajectory points.

The probability density distributions of the background flow fields under the three flow velocity settings were primarily concentrated between 0.20 and 1.23 m/s, with prominent peaks at 0.25–0.50 m/s (6.3%, 5.8%, and 5.5% for flow conditions I, II, and III, respectively). Although the spatial positions of specific velocity zones varied among settings, the overall flow velocity spectrum within the flume remained largely stable, with most velocities falling between 0.30 and 0.50 m/s (Figure 6a). From a behavioral ecology perspective, this moderate-flow range corresponds to energetically favorable conditions for upstream swimming. The consistency of background flow across treatments suggests that observed differences in fish movement patterns are unlikely to result from large-scale changes in overall flow magnitude but instead reflect behavioral responses to local spatial heterogeneity within the flume.

Under the Moderate–High–Low sequence, trajectory points were predominantly distributed within the 0.25–0.45 m/s and 0.50–0.75 m/s intervals, with a pronounced peak at 0.25–0.45 m/s (14.8%). This pattern suggests that individuals preferentially occupied moderate-flow zones when such regions were encountered early along the upstream path. Under the High–Moderate–Low sequence, the probability density distribution exhibited multiple local maxima across the 0.25–0.75 m/s range, with a maximum of 10.3%, indicating a broader exploration of available velocity patches before stabilizing movement paths. In contrast, under the Low–Moderate–High sequence, trajectory points were mainly concentrated within the 0.25–0.51 m/s interval, with only limited use of higher-velocity zones (0.75–1.00 m/s; peak 1.8%). This distribution implies that when low-velocity areas were encountered first, fish tended to maintain occupancy within energetically favorable regions and showed reduced willingness to enter faster-flow patches (Figure 6b). Overall, these sequence-dependent differences suggest that upstream movement patterns were shaped not only by absolute velocity magnitude but also by the spatial arrangement of velocity gradients within the flume.

Under the Low–Moderate–High flow velocity sequence, trajectory points of *L. elongata* in the schooling group were primarily distributed within the 0.20–0.50 m/s and 0.60–0.90 m/s intervals, with moderate peaks at 4.0% and 3.2%, respectively. This pattern suggests that when low-velocity regions were encountered first, schooling individuals tended to maintain collective movement within energetically favorable zones, while only a portion of the group extended into higher-velocity patches. Under the High–Moderate–Low sequence, the probability density distribution showed multiple local maxima within 0.25–0.50 m/s, reaching a maximum of 14.6%. This pronounced clustering within moderate velocities indicates that, despite initially encountering high flow, groups rapidly adjusted their trajectories toward hydraulically favorable regions, reflecting coordinated avoidance of energetically costly areas. In the Moderate–High–Low sequence, trajectory points were mainly concentrated within 0.25–0.60 m/s, with a dominant peak at 0.25–0.40 m/s (9.4%). The narrower concentration around moderate velocities suggests that schooling behavior enhanced collective stabilization within optimal flow zones. Overall, these results indicate that under schooling conditions, upstream movement patterns were strongly biased toward moderate-flow regions, and that spatial velocity sequence influenced how rapidly and cohesively groups adjusted their collective trajectories within the heterogeneous hydraulic environment (Figure 6c).

**Figure 7:**
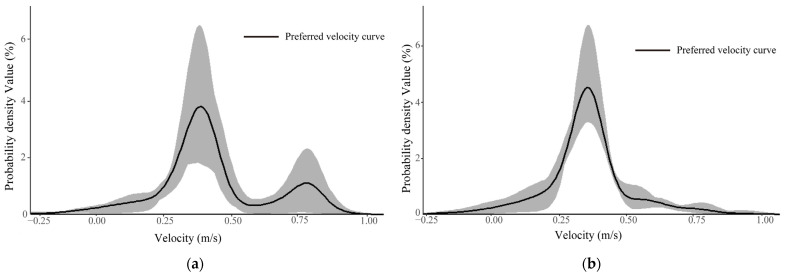

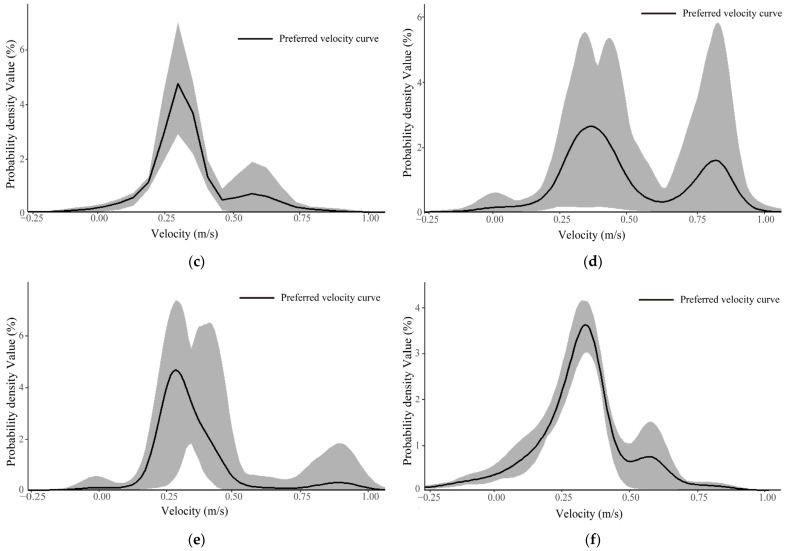
Probability density distribution diagrams of flow velocity under three flow velocity conditions include (**a**) solitary fish Low–Moderate–High preferred curve, (**b**) solitary fish High–Moderate–Low preferred curve, (**c**) solitary fish Moderate–High–Low preferred curve, (**d**) schooling group Low–Moderate–High preferred curve, (**e**) schooling group High–Moderate–Low preferred curve, and (**f**) schooling group Moderate–High–Low preferred curve. The shaded area represents the 95% confidence interval.

In the solitary fish group, condition I produced two preferred velocity ranges (0.25–0.50 m/s and 0.55–0.80 m/s; Figure 7a), suggesting flexible use of both moderate and moderately high flows when hydraulic heterogeneity was pronounced. Under Condition II, preferences were concentrated within 0.21–0.48 m/s (Figure 7b), whereas under Condition III the preferred range shifted toward lower velocities (0.20–0.35 m/s; Figure 7c). The overlap between Conditions I and II (0.25–0.48 m/s) indicates a stable core preference for moderate-flow environments, while the downward shift under Condition III suggests increased selection for energetically conservative strategies when higher velocities became more extensive.

In the three-fish schooling group, Condition I again showed two preferred ranges (0.25–0.50 m/s and 0.60–1.00 m/s; Figure 7d), but the broader upper range implies that collective movement facilitated limited exploitation of faster flows. Under Condition II, preference was strongly concentrated within 0.23–0.50 m/s (Figure 7e), and under Condition III, two adjacent moderate ranges (0.24–0.47 m/s and 0.50–0.60 m/s; Figure 7f) were identified. Across treatments, schooling fish consistently selected velocities between 0.23 and 0.50 m/s, indicating that group cohesion reinforced stabilization within energetically efficient flow zones.

Overall, these results demonstrate that moderate-flow environments (approximately 0.23–0.50 m/s) represent a core preferred range for upstream movement. While solitary individuals showed greater flexibility under certain hydraulic conditions, schooling behavior appeared to buffer environmental variation by promoting collective retention within energetically favorable velocity bands.

### 3.4. Schooling Status

#### 3.4.1. Nearest Neighbor Distance (NND)

The linear mixed-effects model indicated that flow condition had no significant effect on the log-transformed nearest neighbor distance (*logDist*) of schooling *L*. *elongata* (Type II *ANOVA*: *F* = 1.857, *p* = 0.174). Fixed-effect estimates suggested a tendency for logDist to increase under Flow II relative to Flow I (*Estimate* = 0.886, *p* = 0.144) and a slight decrease under Flow III (*Estimate* = −0.130, *p* = 0.826), but neither effect reached statistical significance. Residual diagnostics indicated that normality and homoscedasticity assumptions were reasonably satisfied, and the variance of the FishID random intercept (0.442, *SD* = 0.665) reflected moderate among-individual variation. Post-hoc pairwise comparisons confirmed no significant differences in *logDist* among the three flow conditions (*p* > 0.05).

Biologically, these results suggest that, once repeated measurements of individual fish are accounted for, the spatial heterogeneity of flow does not substantially alter nearest neighbor distances within schools. This indicates that schooling cohesion is maintained across different flow regimes, and fish are able to preserve collective structure regardless of local variations in hydraulic conditions.

#### 3.4.2. Mean Pairwise Distance (MPD)

The linear mixed-effects model indicated that flow condition had a significant effect on the mean pairwise distance (MPD) of three-fish groups (Type III *ANOVA*: *F* = 15.794, *p* < 0.001). Fixed-effect estimates suggested that, compared with Flow I, MPD tended to decrease under Flow II (*Estimate* = −0.205, *p* = 0.068) and increased significantly under Flow III (*Estimate* = 0.362, *p* = 0.004). Residual diagnostics indicated that normality and homoscedasticity assumptions were reasonably satisfied, and the variance of the GroupID random intercept (0.026, *SD* = 0.160) reflected moderate among-group variation. Post hoc pairwise comparisons confirmed that MPD under Flow III was significantly higher than under Flow I and Flow II, whereas the difference between Flow I and Flow II was not statistically significant (*p* > 0.05). Biologically, these results suggest that flow heterogeneity can influence group spacing, with higher flow conditions leading to a more dispersed schooling structure. However, the moderate variation among groups indicates that fish are able to maintain cohesive interactions even under different hydraulic regimes, highlighting the flexible yet stable nature of schooling behavior in *L. elongata*.

## 4. Discussion

### 4.1. Preferred Velocity During L. elongata Upstream Migration

Spatially heterogeneous flow can be characterized by properties such as intensity, periodicity, orientation, and spatial scale, among which mean velocity is a key environmental factor influencing the upstream migration of *L. elongata* [30]. In the present study, under three mean velocity conditions, individuals predominantly occupied low-velocity regions (0.20–0.50 m/s) and preferentially explored even lower-velocity zones during meandering upstream swimming (Table 2). From a behavioral ecology perspective, this pattern likely reflects an energy-conservation strategy, where individuals reduce locomotor costs while maintaining upstream orientation [33,34,35]. Beyond velocity magnitude, heterogeneity in the mean velocity distribution influenced swimming performance. Fish swam significantly faster in high-flow regions than in low- or moderate-flow areas, suggesting behavioral flexibility: individuals actively adjusted swimming speed in response to elevated energetic demands, potentially to reduce exposure to stressful hydraulic conditions [33]. These individual-level responses—selecting low-velocity refuges and accelerating in high-velocity zones—may scale to group-level patterns in schools, affecting cohesion, exploration, and utilization of different flow regions. Across the three flow conditions, *L. elongata* primarily preferred velocities of 0.25–0.48 m/s, with a secondary range of 0.55–0.80 m/s under condition I. This bimodal preference likely reflects individual variability in physiological and behavioral traits, including body size and swimming capacity [36]. Indeed, two larger individuals (>23 cm) and one highly active fish with greater swimming duration may have contributed to this pattern [37]. These observations suggest that individual differences influence habitat selection within heterogeneous flows, which can, in turn, affect group distribution and movement when fish interact socially. Analysis of background velocity distributions indicated that fish preferentially occupied regions with higher spatial availability and lower mean velocities. For example, under condition I, velocities were distributed mainly within 0.20–0.51 m/s and 0.75–1.00 m/s, with fish favoring the more prevalent low-velocity range. This demonstrates that upstream pathway selection is shaped by both environmental heterogeneity and the spatial distribution of hydraulic features, rather than absolute velocity alone. It is important to acknowledge the experimental limitations. Interfaces between regions of differing mean velocity, as well as turbulence and fine-scale hydraulic gradients, were not directly quantified, and the spatial scale of the flume, along with potential effects of captivity and acclimation, may have influenced behavior.

### 4.2. Schooling Behavior on Fish Upstream Migration Efficiency and Velocity Selection

Schooling behavior enhances navigational capability by allowing fish to collectively identify migratory routes, reduce migration time, and increase success rates [20]. In this study, *L. elongata* schools exhibited more regular and aggregated spatial distributions compared to solitary individuals. Comparison of mean upstream migration times revealed that schooling groups migrated significantly faster than solitary fish (*p* < 0.05) (Table 3), suggesting that social attraction and guidance mechanisms facilitate optimized path selection, reduce trajectory deviations, and minimize additional energy expenditure, thereby improving migration efficiency. These benefits are influenced by velocity gradients within the flow field [38,39,40]. At the individual level, once a single fish initiates movement, conspecifics tend to follow by imitating its behavior while maintaining characteristic inter-individual spacing, reflecting the fundamental behavioral rules of repulsion, orientation, and attraction [41,42]. Schooling fish consistently preferred flow velocities of 0.23–0.50 m/s, often occupying low-velocity areas and avoiding high-velocity zones, as shown by background velocity probability density plots [37,43]. This pattern likely arises from a combination of exploratory tendencies and leadership effects of key individuals [44,45]. For instance, individuals occasionally detached from the school to explore other upstream regions, after which the group followed and re-aggregated, consistent with observations in other species that individuals with stronger social connections are more likely to guide group movement [46,47]. Analysis of nearest-neighbor distance (NND) across different heterogeneous flow conditions revealed no significant differences among treatments (*p* > 0.05), indicating that inter-individual spacing and interaction patterns were maintained despite variations in flow. To further assess group-level spatial structure, the mean pairwise distance (MPD) was analyzed. Results showed that MPD increased significantly under Flow III compared to Flow I and II (*p* < 0.01), suggesting that higher flow heterogeneity led to more dispersed group spacing, while overall cohesion (as measured by NND) remained stable. This indicates that although schools maintained basic cohesive structure, individuals adjusted their relative positions in response to localized hydraulic stress, resulting in a more expanded group configuration under highly heterogeneous conditions. In addition to these group-level observations, this study also used first-arrival time as a proxy for upstream migration efficiency, reflecting short-term migration performance. However, it should be noted that first-arrival time may also be influenced by individual behavioral traits, such as exploratory tendency, motivation, and risk-taking [44,45]. Under experimental conditions, variation in this metric may arise from both differences in locomotor capacity and behavioral decision-making processes. Previous studies on migratory fish have also employed similar indicators, such as attraction time [31]. Therefore, while the first-arrival time provides valuable information on migration efficiency, its interpretation should take into account both the physiological and behavioral factors that contribute to this measure. From a behavioral ecology perspective, individual responses to environmental heterogeneity—such as velocity selection, exploratory excursions, and social following—scale up to influence group-level movement patterns, ensuring both efficient upstream navigation and maintenance of group structure [48]. Lei et al. studied the swimming capacity of *Diptychus maculates* steindachner, a benthic migratory fish species in the Muzat River, Xinjiang, which shares similar ecological traits with *L. elongata*. The study recommended a mainstream flow velocity range of 0.20–1.02 m/s for fishway design, with the optimal velocity interval showing strong consistency with the 0.20–0.50 m/s range observed in the present study. This cross-species consistency suggests that benthic migratory fishes may have evolved similar energy-conservation strategies, actively selecting low-velocity zones as hydraulic refuges during migration [49]. Such behavioral traits should be considered a key factor in fishway design.

### 4.3. Limitations and Future Directions

This study provides a systematic analysis of upstream migration efficiency, swimming strategies, preferred flow velocity ranges, and schooling dynamics, offering new insights into the volitional swimming behavior of *L. elongata* under near-natural flow conditions. From a behavioral ecology perspective, the observed patterns demonstrate how individual responses to flow heterogeneity—such as velocity selection, boundary use, and exploratory movement—can scale up to influence group-level outcomes, including cohesion, coordinated upstream navigation, and overall migration efficiency.

However, several limitations should be considered when interpreting these results. Due to the protected status of *L. elongata* as a Class II nationally protected species in China, the number of experimental fish was limited, and the group size was therefore set to three individuals. This group size represents a minimal grouping context and does not necessarily reflect the natural schooling structure of the species. Given that schooling behavior can vary with ecological conditions, season, and behavioral context, caution is required when translating these findings into management strategies. Despite these uncertainties, our results provide valuable insights into the collective swimming behavior of *L. elongata*. In addition, indoor flume experiments inevitably simplify natural river conditions, with confined spaces, relatively uniform flow regimes, and handling procedures potentially influencing individual and collective behavior. These factors may affect swimming performance, energy expenditure, and social interactions, highlighting that the observed behaviors may not fully capture responses in more complex natural habitats.

Future studies should aim to replicate more realistic hydraulic conditions, such as those found in vertical-slot or pool-and-weir fishways, where spatial heterogeneity, velocity gradients, and turbulence structures are more pronounced. The integration of high-resolution flow measurement techniques, such as particle image velocimetry (PIV) and laser Doppler anemometry (LDA), with three-dimensional fish tracking would enable a more detailed understanding of how fine-scale hydraulic features shape individual and collective behaviors. Such approaches could help identify flow thresholds and turbulence regimes that facilitate schooling-assisted passage, thereby supporting biologically informed fishway design.

Importantly, the experimental framework developed in this study provides a useful platform that can be extended to other migratory fish species, including those of ecological or conservation concern in different geographic regions. Comparative studies across species and flow conditions would improve our understanding of how behavioral strategies vary among taxa and environments, ultimately contributing to more generalizable principles for fish passage design and river management.

## Figures and Tables

**Table 1:** Average inlet flow velocity in different flow zones under three flow velocity settings.

Spatially Heterogeneous Flow Velocity	Low Velocity Area (m/s) Mean ± SD	Moderate Velocity Area (m/s) Mean ± SD	High Velocity Area (m/s) Mean ± SD
I (Low–Moderate–High)	0.44 ± 0.11	0.78 ± 0.15	1.16 ± 0.22
II (High–Moderate–Low)	0.47 ± 0.09	0.78 ± 0.11	1.12 ± 0.13
III (Moderate–High–Low)	0.48 ± 0.07	0.75 ± 0.07	1.10 ± 0.09

**Table 2:** Summary of velocity preference and behavioral responses of solitary *L. elongata* under three heterogeneous flow conditions.

Flow Condition	Preferred Velocity Range (m/s)	Secondary Range (If Any)	Key Behavioral Observations
I (Low–Moderate–High)	0.25–0.50	0.55–0.80	Bimodal preference; fish used both moderate and moderately high flows; meandering paths.
II (High–Moderate–Low)	0.21–0.48	None	Concentrated in moderate velocities; avoided high flow despite initial exposure.
III (Moderate–High–Low)	0.20–0.35	None	Shift toward lower velocities; conservative strategy when high flow was extensive.

**Table 3:** Comparison of upstream migration performance and group metrics between solitary and schooling *L. elongata* under different flow conditions.

Parameter	Solitary Fish	Schooling Fish	Statistical Outcome
First-arrival time (s)	Slower	Faster (−8.93 s)	*p* < 0.01 (Condition effect)
Preferred velocity range	0.25–0.48 (m/s)	0.23–0.50 (m/s)	Consistent moderate-flow preference
Wall-following proportion	63.8–80.5%	20.5–26.7%	Schools explored wider channel
Nearest neighbor distance (NND)	—	No significant difference among flows	*p* > 0.05; cohesion maintained
Mean pairwise distance (MPD)	—	Increased under Flow III	*p* < 0.01; more dispersed at high heterogeneity

## Data Availability

The data provided in this study are available upon request from the corresponding author.
